# A sarcopenia screening test predicts mortality among hospitalized cancer patients

**DOI:** 10.14814/phy2.16173

**Published:** 2024-08-05

**Authors:** Wen‐Li Lin, Jyh‐Jou Chen, Li‐Min Wu, Wen‐Tsung Huang, How‐Ran Guo, Thi‐Hoang‐Yen Nguyen

**Affiliations:** ^1^ Department of Medical Affairs Chi Mei Medical Center Tainan Liouying Taiwan; ^2^ School of Nursing Fooyin University Kaohsiung Taiwan; ^3^ Division of Gastroenterology and Hepatology, Department of Internal Medicine Chi Mei Medical Center Tainan Liouying Taiwan; ^4^ School of Nursing Kaohsiung Medical University Kaohsiung Taiwan; ^5^ Adjunct Research Fellow, Department of Medical Research Kaohsiung Medical University Hospital Kaohsiung Taiwan; ^6^ Division of Hematology and Oncology, Department of Internal Medicine Chi‐Mei Medical Center Tainan Liouying Taiwan; ^7^ Department of Environmental and Occupational Health National Cheng Kung University Tainan Taiwan; ^8^ Department of Occupational and Environmental Medicine National Cheng Kung University Hospital Tainan Taiwan

**Keywords:** cancer, hospitalization, mortality, prediction, sarcopenia, screening

## Abstract

This study investigated the ability of a sarcopenia screening test to predict mortality among cancer inpatients. We conducted a prospective study of patients admitted to the oncology ward of a teaching hospital in southern Taiwan. Over a 5‐month period, 82 patients were enrolled for evaluation and were followed for 3 years. All participants received a comprehensive assessment at the time of admission, including Eastern Cooperative Oncology Group (ECOG) performance status, cognitive ability, nutrition index, body mass index, and short physical performance battery (SPPB). Age, ECOG performance status, dementia, SPPB score, and albumin level were associated with sarcopenia. Of the enrolled participants, 53 (64.6%) were diagnosed with sarcopenia. Patients with sarcopenia were associated with worse overall survival (OS) than patients without sarcopenia (28.8% vs. 82%, *p* = 0.01). Metastasis (hazard ratio [HR]: 5.166; 95% confidence interval [CI]: 1.358–19.656) and albumin level (HR: 4.346; 95% CI: 1.493–12.654) were independent and significant predictors of OS for the whole study population. Age was a predictor of 2‐year all‐cause mortality among patients aged ≥65 years but not among those aged <65 years (OS: 25.6% vs. 100%, *p* = 0.04). To summarize, the sarcopenia screening results were found to predict OS and all‐cause mortality and may be helpful for patient stratification during in‐hospital care.

## 1 INTRODUCTION

Sarcopenia is characterized by the age‐associated loss of skeletal muscle mass and is accompanied by reductions in strength and physical performance (Cruz‐Jentoft et al., 2010). Sarcopenia is pathophysiologically associated with various etiologies, including advanced age, lack of exercise, poor nutritional status, inflammatory diseases, and endocrine diseases (Cruz‐Jentoft et al., 2010). Malignant diseases can also cause sarcopenia (Fearon et al., 2011), and sarcopenia has a reported prevalence of 11%–74% among adults diagnosed with cancer, varying according to the definitions employed and the study population of interest.

Sarcopenia, defined as the loss of muscle mass and function associated with aging, and cachexia, defined as weight loss due to an underlying illness, are muscle wasting disorders of particular relevance in the aging population (Ali & Garcia, 2014). The effects of low muscle mass on OS have been observed for various cancer types and across disease stages (Shachar et al., 2016), and sarcopenia has also been associated with poor prognoses in multiple malignancies. Compared with the absence of sarcopenia, the presence of sarcopenia is associated with significantly worse OS among patients with lung cancer, gastrointestinal cancer (Martin et al., 2013; Prado et al., 2008), hepatocellular carcinoma (Harimoto et al., 2013), esophageal cancer (Harada et al., 2016), lymphoma (Go et al., 2016), melanoma (Sabel et al., 2011), and renal cell carcinoma (Fukushima et al., 2016; Psutka et al., 2016). However, the reported associations between sarcopenia and survival among colon cancer patients have been inconsistent (Oh et al., 2020), and the effects of age are seldom evaluated.

Cancer cachexia, also comes with muscle wasting, is a complex metabolic syndrome associated with the underlying illness. It is characterized by severe muscle and fat loss that cannot be fully reversed by conventional nutritional support. Besides severe weight loss (>5% over 12 months or BMI <20 kg/m^2^), systemic inflammation (evidenced by biomarkers such as C‐reactive protein [CRP] or interleukin [IL]‐6 levels) supports a diagnosis of cachexia (Evans et al., 2008; Fearon et al., 2011). In contrast, sarcopenia is primarily a condition of age‐related loss of muscle mass and strength, which can lead to frailty, disability, and other adverse health outcomes (Evans et al., 2008).

The most recent guidelines define low muscle strength as a handgrip strength <28 kg for men and < 18 kg for women. The criteria for low physical performance are a 6‐Meter Timed Walk speed <1.0 m/s, a Short Physical Performance Battery (SPPB) score ≤9, or a Five‐Times Sit‐to‐Stand test ≥12 s. The Asian Working Group on Sarcopenia (AWGS) 2019 guidelines retained the original cutoffs for height‐adjusted muscle mass: bioimpedance <7.0 kg/m^2^ in men and <5.7 kg/m^2^ in women (Chen et al., 2020). The prevalence of sarcopenia varies according to the population studied and the assessment methods used (Oken et al., 1982). The AWGS 2014 guidelines recommended the assessment of appendicular skeletal muscle mass using bioelectrical impedance analysis (BIA) to identify patients with sarcopenia. The updated AWGS 2019 guidelines recommend additional strategies for the early identification of people with or at risk for sarcopenia to facilitate necessary interventions in settings with limited access to advanced diagnostic equipment (Chen et al., 2020).

Newly diagnosed cancer cases are expected to reach 21.4 million globally by 2030 (Pfeiffer, 1975). In Taiwan, cancer has been the leading cause of death for decades. Identifying clinical factors associated with increased risks of cancer progression and mortality is of uttermost importance for improving prognostic estimations and determining appropriate treatment strategies (Pamoukdjian et al., 2018). Cancer patients with sarcopenia typically have a poor prognosis and are more likely to develop complications associated with cancer treatment (World Health Organization, 2020). In addition to the prevalence of sarcopenia, the risk factors associated with sarcopenia incidence are also worth exploring in cancer patients. The primary objective of this study was to use a sarcopenia screening test to identify sarcopenia in hospitalized patients with cancer at hospital admission and thus estimate its prevalence and investigate the ability of the screening to predict mortality among those inpatients.

## 2 MATERIALS AND METHODS

### 2.1 Study subjects

**FIGURE 1:**
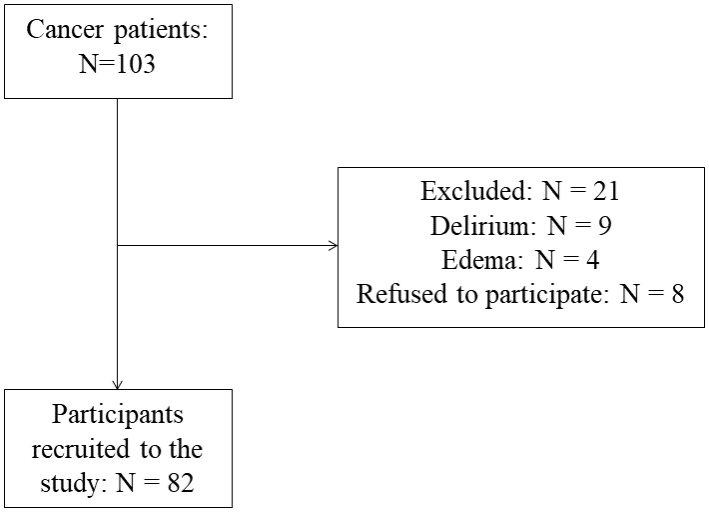
Flow chart of the selection of study participants.

This study was approved by the institutional Ethics of Research Committee of Chi Mei Medical Center, Taiwan (10907‐L03). The protocol conformed to ethical standards according to the Declaration of Helsinki of 1975, as revised in 2008. All patients with cancer (*n* = 103) who were consecutively admitted to participating wards between August 2020 and December 2020 were entered into the study protocol. Survival status was assessed at 12 and 24 months after the baseline investigation. Patients with the following conditions at the time of admission were excluded: (1) severe cognitive impairment; (2) delirium; and (3) clinically visible edema. The only exclusion criterion was an unwillingness to participate in the study. Eight patients were unable to follow study procedures, and 13 participants had delirium or edema and were not further assessed. The final sample included 82 participants. Twenty‐two patients received surgery plus chemotherapy, while 60 patients received chemotherapy alone (Figure 1). All the patients provided their written informed consent to participate in this study.

### 2.2 Sarcopenia screening

The AWGS 2019 consensus defines sarcopenia as “age‐related loss of muscle mass, plus low muscle strength, and/or low physical performance.” (Chen et al., 2020). The skeletal muscle mass index (SMI, in kg/m^2^) is a measure of relative muscle mass that can be used to differentiate between lean mass components, such as organs and muscle (da Cunha et al., 2019). Muscle mass was assessed by measuring SMI using BIA (InBody 230; Biospace Co. Ltd, Seoul, Korea). Low muscle mass was defined based on cutoff points of BIA <7.0 kg/m^2^ for men and BIA <5.7 kg/m^2^ for women. The AWGS 2019 guidelines recommend using BIA to measure muscle mass when diagnosing sarcopenia (Evans et al., 2008). Muscle strength was assessed by measuring handgrip strength (HGS) using a hydraulic‐type Jamar dynamometer, which is often used in Asia for this purpose (World Health Organization, 2020). The patients used the hand dynamometer for 5 s on each hand, and the result from the strongest hand was used for analyses. Low muscle strength was defined as handgrip strength <28 kg for men and < 18 kg for women. The criteria for low physical performance were a 6‐Meter Walk Test speed <1.0 m/s and an SPPB score ≤9 (Chen et al., 2020).

### 2.3 Measures

Each patient was subjected to a clinical examination, and abdominal and pelvic computed tomography (CT) scans were obtained prior to surgery. The variables assessed included patient age, sex, tumor–node–metastasis (TNM) stage, personal medical history, comorbidities (hypertension, congenital heart defects [CHD], congestive heart failure [CHF], chronic obstructive pulmonary disease [COPD], diabetes, stroke), Eastern Cooperative Oncology Group (ECOG) performance status, selected blood count and biochemical parameters (i.e., albumin, hemoglobin), Charlson Comorbidity Index, and length of hospital stay. We also evaluated the participants for performance on Activities of Daily Living (ADL), Short Portable Mental Status Questionnaire (SPMSQ), and SPPB, and the number of drugs used.

The ECOG Performance Status Scale was designed to assess the level of functioning of patients with cancer in terms of their ability to care for themselves, daily activity, and physical ability (Oken et al., 1982). In addition, the SPMSQ is a brief questionnaire designed to screen for cognitive impairment, often used in elderly patients, consisting of 10 simple questions that test orientation, memory, and mathematical ability, including the patient's awareness of current events, personal history, and ability to perform simple calculations (Pfeiffer, 1975).

### 2.4 Statistical analysis

Descriptive statistics for categorical variables are presented as the absolute number and frequency, and normally distributed continuous variables are presented as the mean ± standard deviation (SD). Fisher's exact test and the Chi‐square test were used to compare categorical variables. Data analyses were performed using SPSS version 26.0 (SPSS Inc, Chicago, IL, USA), with *p* < 0.05 indicating significance. We performed survival analysis using univariate and multivariate Cox proportional hazard models to calculate the hazard ratio (HR) and 95% confidence interval (CI) of factors associated with sarcopenia incidence. Covariates that were identified as significant in the univariate Cox models (*p* < 0.05) were included in the multivariate Cox model. In addition, survival curves were estimated using the Kaplan–Meier method and compared using log‐rank tests.

## 3 RESULTS

### 3.1 Patient characteristics

A total of 103 participants were enrolled in the baseline investigation; 21 patients were excluded from the study due to delirium (nine individuals), edema (four individuals), or refusal to participate (eight individuals). As a result, 82 participants (29 women, 53 men; mean age: 62.72 ± 10.7 years) were included in the baseline analyses (Figure 1). Of these, 53 (64.6%) participants were diagnosed with sarcopenia. Table 1 shows the baseline characteristics of participants according to sarcopenia status. The mean age of the sarcopenia group was higher than that of the non‐sarcopenia group (*p* < 0.01). Common comorbidities were similar between the sarcopenia and non‐sarcopenia groups. Significant differences in ECOG performance status (*p* = 0.001), SPMSQ score (*p* = 0.001), and serum albumin level (*p* = 0.001) were observed between the sarcopenia and non‐sarcopenia groups. In addition, the handgrip strength (*p* = 0.001) and SPPB score (*p* = 0.001) were significantly lower in the sarcopenia group than in the non‐sarcopenia group.

**TABLE 1:** Selected general characteristics and comorbidities of study participants according to the incidence of sarcopenia.

		Incidence of sarcopenia		
	Total sample (*n* = 82)	No (*n* = 29)	Yes (*n* = 53)	*p*‐value
Age (years), mean ± SD	62.72 (10.70)	57.28 (8.94)	65.70 (10.48)	0.001
≥65	38 (46.34)	6 (20.69)	32 (60.38)	0.001
<65	44 (53.67)	23 (79.31)	21 (39.62)	
Female, *n* (%)	29 (35.4)	12 (41.4)	17 (32.1)	
Cancer type				
Colon cancer	25 (30.4)	9 (32.2)	16 (29.6)	0.876
Breast cancer	9 (10.9)	4 (14.3)	5 (9.3)	
Head and neck cancer	20 (24.4)	6 (21.4)	14 (25.9)	
Hepatocellular carcinoma	12 (14.7)	3 (10.7)	9 (16.7)	
Lung cancer	6 (7.4)	2 (7.1)	4 (7.4)	
Other	10 (12.2)	4 (14.3)	6 (11.1)	
BMI (kg/m^2^), mean ± SD	23.86 (3.62)	23.92 (2.04)	23.83 (4.26)	0.914
BMI				
<21	17 (20.73)	2 (6.90)	15 (28.30)	0.045
21–24	34 (41.46)	16 (55.17)	18 (33.96)	
>24	31 (37.80)	11 (37.93)	20 (37.74)	
SPPB				
≤9	51 (62.20)	0 (0.00)	51 (96.23)	0.001
>9	31 (37.80)	29 (100.0)	2 (3.77)	
ECOG				
0–1	29 (35.37)	29 (100.0)	0	0.001
2–3	53 (64.63)	0 (0.0)	53	
Stage				
Non‐metastasis	40 (48.7)	21 (72.4)	19 (35.8)	0.002
Metastasis	42 (51.3)	8 (27.6)	34 (64.2)	
SPMSQ				
Normal–mild cognitive impairment	65 (79.27)	29 (100.0)	36 (67.92)	0.001
Moderate–severe cognitive impairment	17 (20.73)	0 (0.0)	17 (32.08)	
Sarcopenia index				
Handgrip strength mean ± SD	21.40 ± 7.73	25.30 ± 9.35	19.27 ± 5.75	0.001
SPPB mean ± SD	7.95 ± 3.39	11.69 ± 2.38	5.91 ± 1.68	0.001
SMI (kg/m^2^), mean ± SD				
Low (male <7.0; female <5.7)	21 (25.6)	10 (34.5)	11 (20.7)	0.194
Normal	61 (74.4)	19 (65.5)	42 (79.3)	
SMI, mean ± SD	7.3 ± 1.7	7.4 ± 1.7	7.3 ± 1.8	0.112
SPMSQ, mean ± SD		0.34 (0.55)	1.08 (1.37)	0.008
Hypertension, *n* (%)		9 (31.03)	22 (41.54)	0.4755
CHD, *n* (%)		2 (6.9)	4 (7.55)	0.914
Diabetes, *n* (%)		4 (13.79)	18 (33.96)	0.068
COPD, *n* (%)		1 (3.45)	3 (5.66)	0.657
Stroke, *n* (%)		0 (0.0)	2 (3.77)	0.290
Second cancer, *n* (%)		28 (96.55)	52 (8.11)	0.661
Serum albumin, mean ± SD	3.50 (0.72)	3.83 (0.56)	3.32 (0.74)	0.001
Serum albumin				
≤3.5	32 (39.02)	4 (13.79)	28 (52.83)	0.001
>3.5	50 (60.98)	25 (86.21)	25 (47.17)	
Neutrophil lymphocyte ratio				
≤3.0	61 (74.3)	27 (93.1)	34 (64.1)	0.004
>3	21 (25.7)	2 (6.9)	19 (35.9)	
Hemoglobin, mean ± SD	11.84 ± 1.68	11.77 ± 1.56	11.88 ± 1.75	1.00
≤12	48 (58.54)	17 (58.62)	31 (58.49)	
>12	34 (41.46)	12 (41.4)	22 (41.5)	
Length of hospital stay (days), mean ± SD	4.78 ± 3.09	4.41 ± 4.03	4.98 ± 2.45	0.430

Abbreviations: BMI, body mass index; CHD, coronary heart disease; CHF, congestive heart failure; COPD, chronic obstructive pulmonary disease; ECOG, Eastern Cooperative Oncology Group; SD, standard deviation; SMI, skeletal muscle index; SPMSQ, short portable mental status questionnaire; SPPB, short physical performance battery.

### 3.2 Survival analyses

The univariate Cox proportional hazard analysis for OS indicated that sarcopenic patients 65 years and older had a higher mortality risk than their non‐sarcopenic counterparts, but the difference was not significant (HR: 1.77; 95% CI: 0.422–2.392). Sarcopenic individuals were associated with a worse OS than those without sarcopenia (HR: 3.207; 95% CI: 1.130–9.102), and SPPB score (HR: 3.407, 95% CI: 1.209–9.597), ECOG performance status, (HR: 3.207, 95% CI: 1.130– 9.102), and albumin levels (HR: 5.264, 95% CI: 2.059–13.455) were identified as significant predictors for OS (Table 2). The multivariate Cox proportional hazard analysis identified metastasis (HR: 5.166; 95% CI: 1.358–19.656) and albumin levels (HR: 4.346; 95% CI: 1.493–12.654) as independent and significant predictors of OS for the whole study population (Table 3).

**TABLE 2:** Univariate analysis of factors associated with overall survival.

Factor	Overall survival		
	Event	Hazard ratio	95% confidence interval
Age (years)			
≥65	38	1.004	0.422–2.392
<65	44		
SPPB			
Low SPPB score			
≤9	51	3.407*	1.209–9.597
>9	31		
ECOG			
0–1	29	3.207*	1.130–9.102
2–3	53		
Stage			
Non‐metastasis	63	6.723*	1.984–22.781
Metastasis	19		
Diabetes			
Yes	22	2.091	0.903–4.841
No	60		
Serum albumin			
≤3.5	32	5.264**	2.059–13.455
>3.5	50		
Neutrophil lymphocyte ratio			
>3	17	1.128	0.415–3.061
≤3.0	5		
SPMSQ			
Normal–mild cognitive impairment	65	1.320	0.382–4.562
Moderate–severe cognitive impairment	17		
Sarcopenia			
Yes	29	3.207*	1.130–9.102
No	53		

Abbreviations: ECOG, Eastern Cooperative Oncology Group; SPMSQ, short portable mental status questionnaire; SPPB, short physical performance battery.

* *p* < 0.05.

** *p* < 0.01.

**TABLE 3:** Multivariate analysis of factors associated with overall survival.

Factor	Hazard ratio	95% confidence interval
SPPB≤9	3124.55	0.000–3.093
Sarcopenia	3510.39	0.000–3.479
Metastasis	5.166*	1.358–19.656
Serum albumin ≤3.5	4.346*	1.493–12.654

Abbreviation: SPPB, short physical performance battery.

* *p* < 0.05.

**FIGURE 2:**
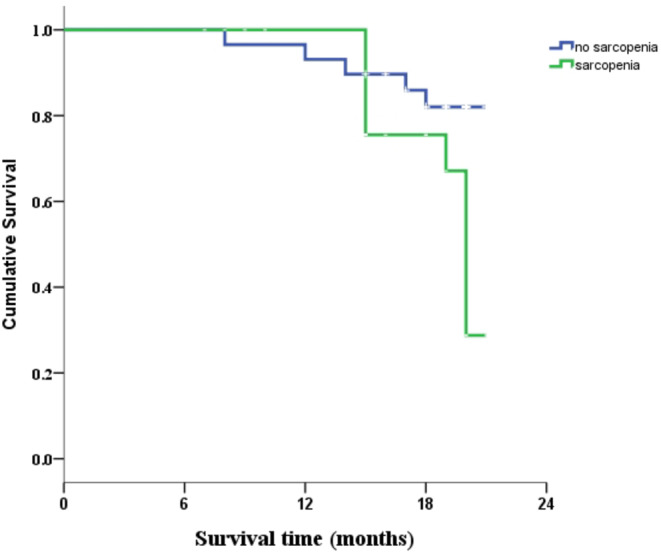
Overall survival of patients with and without sarcopenia.

The Kaplan–Meier survival curves comparing subjects with and without sarcopenia are presented in Figure 2, and the log‐rank test revealed significant differences in survival between the two groups (*p* = 0.01). Mean survival time in the sarcopenia group (18.979 ± 0.365) was shorter than that in the non‐sarcopenia group (19.733 ± 0.580).

**FIGURE 3:**
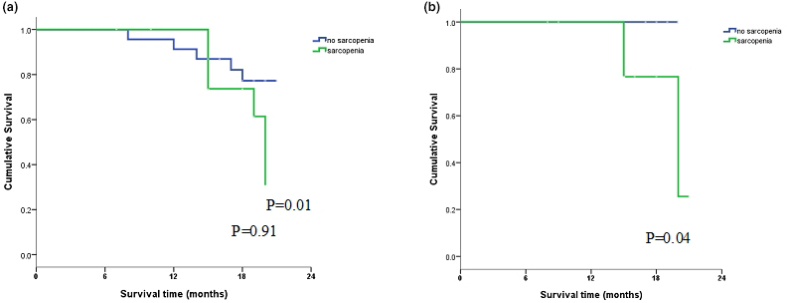
Overall survival of patients with and without sarcopenia aged <65 years (a) and ≥65 years (b).

Subgroup analyses found similar results for patients ≥65 years and those <65 years; however, age was a predictor for 2‐year all‐cause mortality for patients ≥65 years but not for those <65 years (OS: 25.6% vs. 100%, *p* = 0.04; Figure 3).

## 4 DISCUSSION

Sarcopenia is described by the 2013 AWGS consensus guidelines as the “age‐related loss of muscular mass, reduced muscle strength, and/or low physical performance.” The updated AWGS 2019 guidelines retain the prior definition of sarcopenia and establish cutoff values for low muscle strength, including handgrip strength and SPPB scores (Chen et al., 2020). The present study findings showed that patients in the sarcopenia group were older, with higher rates of low handgrip strength and low SPPB scores, compared with patients in the non‐sarcopenia group. Our findings were consistent with the findings of a previous study (Phu et al., 2020). SPPB has been found to have acceptable value for the diagnosis of older adults with sarcopenia. Furthermore, low SPPB scores may be used as a sarcopenia screening tool in clinical settings where appendicular lean body mass measurements are not available.

Sarcopenia has been identified as an independent prognostic factor for a variety of cancer types, such as colorectal cancer (da Cunha et al., 2019), ovarian cancer (Chae et al., 2021), gastrointestinal cancer (Prado et al., 2008), and esophageal cancer (Harada et al., 2016), implying an association between sarcopenia and a shorter life expectancy. When other prognostic variables were controlled for, the current study found no significant association between sarcopenia and OS; however, the Kaplan–Meier and univariate regression analyses suggested a trend toward shorter survival. The effect of sarcopenia is likely significantly weaker than the effects of other strong prognostic indicators, such as serum albumin. Furthermore, when analyzing a larger population, the impact of sarcopenia on OS may become more apparent, which could explain why prior research using very small study populations failed to identify a link between sarcopenia and survival. Our study supports that sarcopenia may be useful for predicting mortality among older adult inpatients.

A systematic review of 35 articles reported a sarcopenia prevalence of 10% among community‐dwelling participants when applying the AWGS definition of sarcopenia (Shafiee et al., 2017). Previous studies examining sarcopenia among hospitalized older adults in acute geriatric wards reported prevalences ranging from 25% to 28% when using the European Working Group on Sarcopenia in Older People (EWGSOP) definition (Rossi et al., 2014). The prevalence of sarcopenia in our study was significantly higher (64.6%), similar to the overall mean probability of sarcopenia of 60% reported by a prospective study conducted among older adults in an in‐hospital rehabilitation setting (Morandi et al., 2015). Our finding that sarcopenia was an independent predictor of long‐term mortality among hospitalized older patients was also consistent with the findings of a previous study (Cerri et al., 2015). In our study, sarcopenic individuals were associated with worse OS than those without sarcopenia (HR: 3.207; 95% CI: 1.130–9.102), similar to a prospective study that found sarcopenia to be independently associated with increased all‐cause mortality (HR: 1.43; 95% CI: 1.09–1.87) (Mayr et al., 2018).

Several potential explanations may underlie the relationship between sarcopenia and poor prognosis. Sarcopenia is more common among patients with >2 metastatic sites (HR: 2.09, 95% CI: 1.24–3.53) (Sharma et al., 2015), and metastasis was identified as a significant and independent predictor of OS in the current study population (HR: 5.166; 95% CI: 1.358–19.656). Moreover, nutritional disorders are common among older adults and might exacerbate the age‐related physiological decline in muscle mass. In addition, lower albumin levels have been identified as a significant, independent factor in prognosis (odds ratio: 2.6, 95% CI: 1.2–5.8) (Sharma et al., 2015), and albumin was identified as a significant and independent predictor of OS in the current study population (HR: 4.346; 95% CI: 1.493–12.654).

The EWGSOP published a consensus paper in 2010 defining sarcopenia in older people based on clinical measures (Fearon et al., 2011). Our study also revealed an association between sarcopenia incidence and mortality among individuals 65 years or older but not among those younger than 65 years (2‐year OS rate: 25.6% vs. 100%, *p =* 0.04). This result was similar to those reported by a prospective study, in which older adult patients with sarcopenia had worse survival than a non‐sarcopenic cohort (“5‐year OS rate” 26.5% vs. 56.0%) (Nakashima et al., 2018).

One limitation of our study should be considered when evaluating our findings. We used the SPPB cutoff threshold of 9 established by the AWGS 2019 guidelines; however, no consensus criteria for a sarcopenia diagnosis have been established across studies. For example, one study set the SPPB cutoff value at 8 (Phu et al., 2020), whereas another study set the cutoff value at 11 (Lee et al., 2021). A review study reported that an SPPB score of less than 10 predicts all‐cause death (Pavasini et al., 2016). Another limitation is that while it is important to differentiate sarcopenia from normal age‐related declines in muscle mass and function, the AWGS 2019 guidelines can not fully achieve this. In fact, we could not differentiate pre‐existing sarcopenia from cachexia, neither. However, because Taiwan is a part of Asia and the AWGS 2019 guidelines are widely used in clinical practice and research, for comparison with other studies in Asia, we believe the results are more compatible by using these guidelines.

## 5 CONCLUSIONS

Our study highlights sarcopenia as a significant concern in older adult inpatients, with a high prevalence and a clear association with worse OS, particularly among those with additional risk factors such as metastasis and low albumin levels. These findings underscore the importance of early detection and management of sarcopenia to potentially improve the outcomes of hospitalized older adults. Sarcopenia's role as an independent prognostic factor for various cancer types further supports its inclusion in comprehensive patient assessments. Future research should aim to refine diagnostic criteria and explore interventions that could mitigate the impact of sarcopenia on morbidity and mortality. Our study contributes to the growing body of evidence indicating that sarcopenia is not just a marker of frailty but a critical factor in patient prognosis and, therefore, deserves greater attention in the clinical management of older adults with cancer and other chronic conditions. Future research that could differentiate pre‐existing sarcopenia from cachexia and differentiate sarcopenia from normal age‐related declines in muscle mass and function, such as those that incorporate the loss of type II motor units into the definition, are needed.

## AUTHOR CONTRIBUTIONS

WLL conceptualized the study, analyzed the data, performed the investigation, drafted the manuscript, critically revised the manuscript; LMW: conceptualized the study, analyzed the data, performed the investigation, drafted the manuscript, critically revised the manuscript; WTH conceptualized the study, analyzed the data, performed the investigation, drafted the manuscript, critically revised the manuscript; HRG conceptualized the study, analyzed the data, performed the investigation, drafted the manuscript, critically revised the manuscript, supervised the study, and validated the data; THYN drafted the manuscript and critically revised the manuscript. JJC analyzed the data, drafted the manuscript, and critically revised the manuscript.

## FUNDING INFORMATION

This study was funded by Chi Mei Medical Center, Liouying [Research Grant CLFHR 10940 and 110CM‐KMU‐03].

## CONFLICT OF INTEREST STATEMENT

The authors declare that the research reported here was conducted in the absence of any commercial or financial relationships that could be construed as a potential conflict of interest.

## ETHICAL APPROVAL AND CONSENT TO PARTICIPATE

This study was approved by the institutional Ethics of Research Committee of Chi Mei Medical Center, Taiwan (10907‐L03). The protocol conformed to ethical standards according to the Declaration of Helsinki published in 1964. All the participants consented to participate in this study.

## CONSENT FOR PUBLICATION

All the participants consented to have their data published.

## Data Availability

The data that support the findings of this study are available from the corresponding author upon reasonable request.
